# Reduction of venous air embolism in coronary computed tomography angiography using a modified method of saline test injection

**DOI:** 10.1186/s12880-023-01006-5

**Published:** 2023-04-11

**Authors:** Weiling He, Feng Huang, An Xie, Xiang Chen, Wenjie Sun, Rui Hu

**Affiliations:** 1 Department of Radiology, Hunan Provincial People’s Hospital (The First Affiliated Hospital of Hunan Normal University), No. 61, West Jiefang Road, Changsha, 410002 Hunan China; 2 Department of Interventional Vascular Surgery, Hunan Provincial People’s Hospital (The First Affiliated Hospital of Hunan Normal University), Changsha, 410002 Hunan China

**Keywords:** Coronary computed tomography angiography, Venous air embolus, Contrast agent, Computed tomography, A modified method of saline test injection

## Abstract

**Objectives:**

This paper analyzed the feasibility of reducing venous air emboli introduced during tube connection in computed tomography angiography (CTA) through a modified method of saline test injection.

**Methods:**

A total of 386 cases of patients undergoing coronary CTA examination were randomly arranged into a control group (199 patients underwent conventional saline injection before the CTA examination) and a case group (187 patients underwent modified saline injection before the CTA examination). The two groups were compared for the location (Fisher’s exact test), number (χ^2^ test), and diameter (Mann-Whitney rank sum test) of the air emboli along the inflow direction of contrast agent within the scan.

**Results:**

The occurrence rate was 10.55% in the control group and 3.74% in the case group respectively, with a statistically different significance (*P* = 0.010). In the case group, there were 7 cases of small-grade venous air emboli. In the control group, there were 15 cases of small-grade venous air emboli and 6 cases of moderate-grade venous air emboli. No cases of large-grade venous air emboli were found in both groups.

**Conclusions:**

The use of this modified method of saline test injection before CTA examination is able to effectively decrease the occurrence of venous air emboli introduced during tube connection, which has some certain practical significance.

## Introduction

Venous air embolism (VAE) is a potentially life-threatening complication of intravenous injection or traumatic vascular injury [1–3], and the embolus that causes VAE is known as a venous air embolus. With the widespread use of computed tomography angiography (CTA) and high-pressure injectors, the occurrence rate of venous air emboli in CTA examination has been reported to be as high as 7-23% [3–5], which may cause the occurrence of VAE [6]. Various factors (such as the volume of introduced air, the speed and geometry of bubbles, the presence of a right to left shunt, and the baseline cardiac function of patients) affect the clinical consequences of VAE, and venous air emboli may result in circulatory col-laps, endothelial damage, cytokine release, microthrombosis, and even tissue ischemia [7].

The currently used high-pressure injectors are mostly equipped with air sensing systems that can efficiently forewarn or halt in case of the existence of air within their tubes, thus preventing air from entering the body. However, the air generated during external tube connection may not be effectually exhausted by the conventional method of saline test injection. The injection processes are hard to be observed by technicians, nurses, and patients, thus making it inconspicuous whether there are air bubbles entering the body of patients during the injection with contrast agent [8]. Even though a small amount of air emboli cannot give rise to adverse consequences, the mental stress of patients may be increased due to cognitive level bias, thus contributing to the tension and panic of patients and sometimes even doctor-patient disputes. In this case, great attention has been attracted to the methods of reducing and preventing the appearance of air bubbles in clinical work [9, 10]. However, the vast majority of air emboli generated during CTA examination are not treated, which creates hidden trouble for CTA examination.

Therefore, it is practically significant to probe methods of decreasing the occurrence of air emboli during the injection of contrast agents. Nevertheless, few Chinese references report the specific solutions for repressing the generation of venous air emboli during CTA examination. Hence, this research discussed the feasibility of decreasing the occurrence of venous air emboli introduced during tube connection through the use of a modified method of saline test injection, with coronary CTA (CCTA) as an example.

## Materials and methods

### Ethics approval and consent to participate

The present research was ratified by the Ethics Committee of Hunan Provincial People’s Hospital and was conducted in accordance with the *Declaration of Helsinki*. All patients had received a questionnaire on injection of iodine contrast agent to ensure no contraindications of intravenous injection with iodine contrast agent and signed the informed consent form.

### Participants

**Fig. 1:**
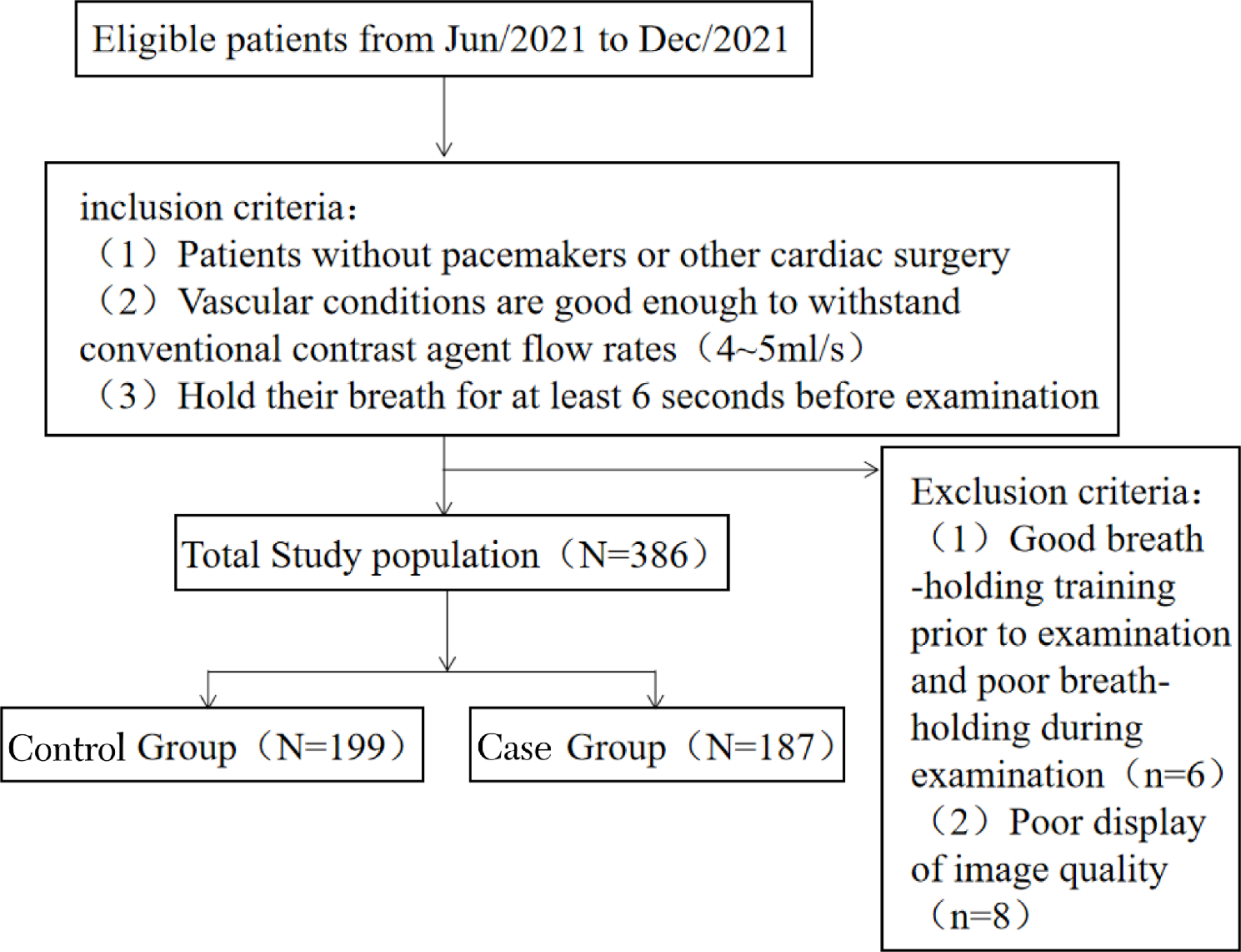
Inclusion and exclusion criteria

The adult patients aged 18–88 years who received CCTA examination in Hunan Provincial People’s Hospital (The First Affiliated Hospital of Hunan Normal University) from June 2021 to December 2021 were included in the research. The inclusion criteria were as follows: (1) patients had not undergone cardiac pacemaker implantations or other cardiac surgeries; (2) patients had a favorable vascular condition and were capable of tolerating the flow rate (4–5 mL/s) of conventional contrast agents; (3) patients were able to hold their breaths for at least 6 s in the training before examination. The exclusion criteria were listed as follows: (1) patients had excellent breath-holding training outcomes before examination and poor breath-holding outcomes during examination; (2) patients had poor-quality images (poor display of superior vena cava, right atrium, right ventricle, or main pulmonary artery structure). A total of 200 cases of eligible patients were chosen to undergo modified saline test injection before CTA examination (the case group) and 200 cases of patients in the same period were randomly selected to receive conventional saline test injection before CTA examination (the control group). Among them, 6 cases were excluded for poor breath-holding results during examination despite excellent breath-holding training outcomes before examination, and 8 cases were excluded for the poor-quality image. Finally, this research included 187 cases in the case group and 199 cases in the control group, a total of 386 patients (Fig. 1).

### Nursing procedures

**Fig. 2:**
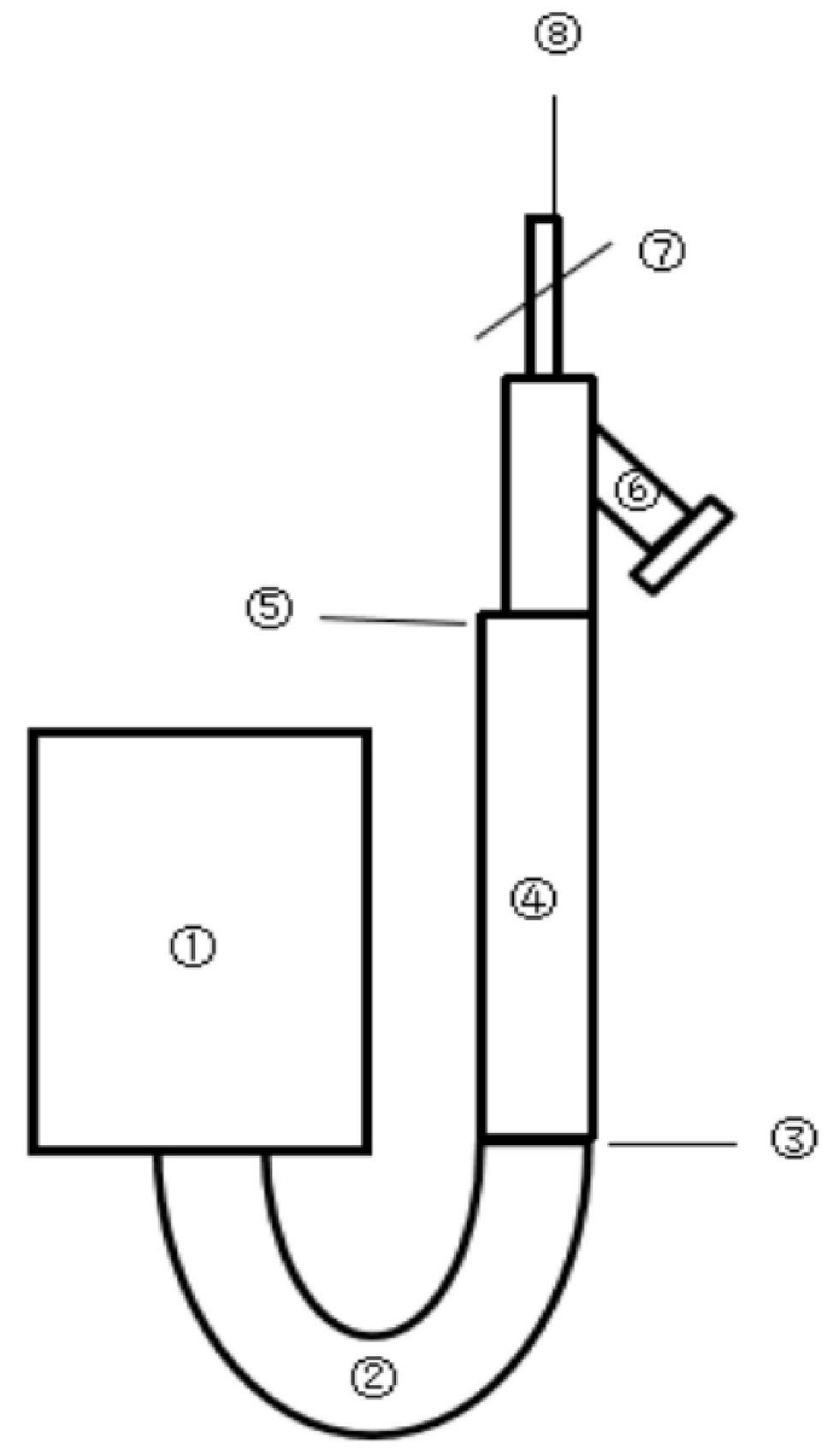
Diagram for the conventional saline test injection Notes: ① The high-pressure injector; ② The external tube of the high-pressure injector; ③ The connection between the external tube and the disposable pressure extension tube of the high-pressure injector; ④ The disposable pressure extension tube; ⑤ The connection between the disposable pressure extension tube and the Y-type indwelling needle; ⑥ The unconnected outlet of the Y-type indwelling needle; ⑦ The clipping switch for the Y-type indwelling needle; ⑧ The entry of the Y-type indwelling needle into the human body. In the conventional saline test injection, the injection can be conducted after connection in ③ and the open of the clip switch in ⑦

Conventional saline test injection was conducted with the following procedures. (1) As per the instructions, the Y-type 18 G indwelling needle was connected to a disposable pressure extension tube at one end, and the lumen was filled with normal saline. Afterward, the indwelling needle was inserted into the right median cubital vein of patients. (2) After lid removal and disinfection, the normal saline and contrast agent bottles were respectively connected to the NaCl cylinder and the CA1 or CA2 cylinder of the high-pressure injector (CT injector, XD 2000, Mississippi ™). (3) Air in the NaCl cylinder, CA1 or CA2 cylinder, and connecting tube was evacuated successively, after which the heparin cap at one side of the disposable pressure extension tube was removed and the pressure extension tube was connected to the distal end of the connecting tube. Then, we ensured that each part was firmly connected and no air bubbles existed within all tubes. (4) Patients lay in the scanning bed and positioned. Afterward, the Y-type 18 G indwelling needle in the elbow was connected to the high-pressure injector and 15–20 mL normal saline was injected 1–2 times at a flow rate of 2 mL/s to ensure the fluent injection for the following CTA examination [11] (Fig. 2).

**Fig. 3:**
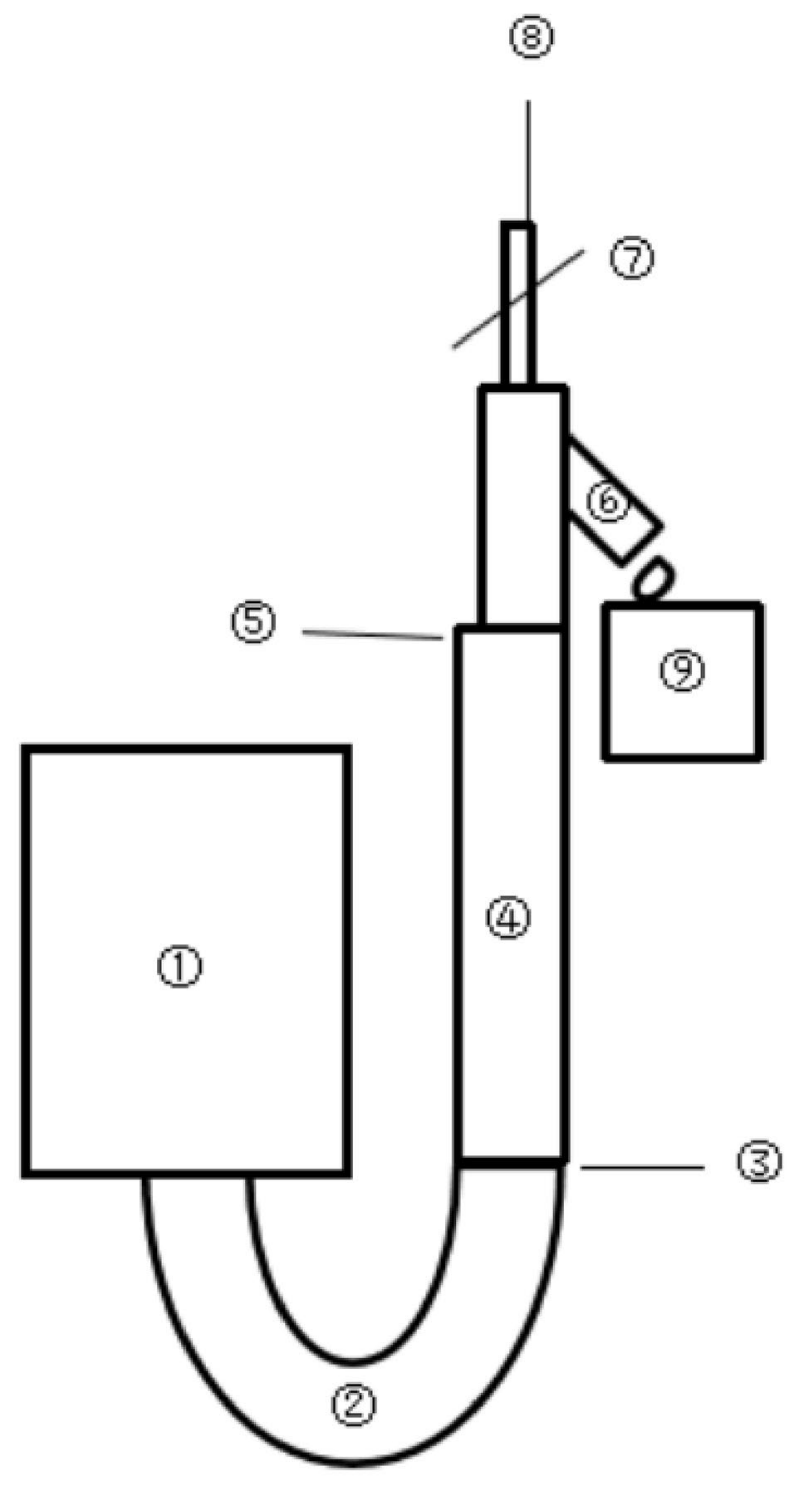
Diagram for the modified saline test injection Notes: ① The high-pressure injector; ② The external tube of the high-pressure injector; ③ The connection between the external tube and the disposable pressure extension tube of the high-pressure injector; ④ The disposable pressure extension tube; ⑤ The connection between the disposable pressure extension tube and the Y-type indwelling needle; ⑥ The unconnected outlet of the Y-type indwelling needle; ⑦ The clipping switch for the Y-type indwelling needle; ⑧ The entry of the Y-type indwelling needle into the human body; ⑨ The three joint design of the Y-type indwelling needle utilized for liquid drainage out of the unconnected outlet. In the modified saline test injection, the injection can be conducted after connection in ③, the close of the clip switch in ⑦, the open of the unconnected outlet in ⑥, and the operation in ⑨

Modified saline test injection was performed. Specifically, the initial operations were the same as the 1–3 steps of the conventional saline test injection. After the completion of step 3, the patient lay in the scanning bed and was positioned. Next, the 18 G indwelling needle in the elbow was clipped, whose disposable pressure extension tube was then connected to the high-pressure injector. Liquids (20–30 mL) were drained out of the unconnected outlet (to exhaust the probably generated air in the tubes during connection) with the use of the three joint design of the indwelling needle. After drainage, the heparin cap was used to close the drainage outlet and the clipped 18 G indwelling needle was opened. Subsequent to all of the aforesaid operations, saline test injection (15–20 mL normal saline was injected 1–2 times at a flow rate of 2 mL/s) was conducted to ensure the fluent injection of normal saline, followed by CTA examination (Fig. 3).

### CCTA examination

All of the participants underwent CCTA on a SIEMENS Force Dual-source Instrument with prospective electrocardiogram gating. The scanning range covered the whole heart and coronary artery from the tracheal bifurcation to the diaphragmatic surface level at the cardiac base. The scanning methods were as follows: after plain scanning, the scanning was enhanced with the contrast agent trace method and an area-of-interest region (the ascending aorta) was set with a threshold value of 100 Hu; when the threshold value was reached, the scanning was automatically trigged. The scanning parameters included automatic tube voltage, automatic detector width, the acquisition layer of 0.75 mm, the interlayer spacing of 0.4 mm, the rotating time of 0.25 s, and the mean time to scan a whole heart of 3–6 s. The median cubital vein was injected with 370 mgI/mL iopromide (ultravist, Bayer AG, Leverkusen, Germany) or 350 mgI/mL iohexol (General Electric Company, Fairfield, Connecticut, USA) at a flow rate of 4–5 mL/s or with 50–60 mL normal saline. After examination, the patient was advised to stay for 0.5-h observation. Patients left without discomfort.

### Image analysis

After scanning, data were input into the picture archiving and communication system of Hunan Provincial People’s Hospital (The First Affiliated Hospital of Hunan Normal University) for image evaluation. The main observations were as follows: the presence of any clear gas shadows along the direction of venous inflow (superior vena cava, right atrium, right ventricle, and main pulmonary artery) within the examination area and the location, number, and diameter of the shadows. The air emboli were graded, and their diameter was measured according to the methods of Groell et al. [12]: small grade, less than 3 air emboli with a diameter of less than 1 cm; moderate grade, over 3 air emboli with a diameter of less than 1 cm or the single air embolus with a diameter of l-2 cm; large grade, the single air embolus with a diameter of over 2 cm or an air-liquid surface with a diameter of 1–2 cm.

### Statistical analysis

All data were processed with SPSS 17.0 statistical software. Measurement data were presented as ratio/percentage, and count data as mean ± standard deviation. The *t*-test was utilized for comparisons of mean ages between two groups, and χ2 for comparisons of gender and the existence of air emboli or not between two groups. The Fisher’s exact test was employed to compare the grade, number, and location of air emboli between two groups, and the Mann-Whitney rank-sum test to compare the diameter of air emboli between two groups. A difference with *P* < 0.05 was considered statistically different between two groups.

## Results

### Basic information

There were 167 males and 219 females among the included patients who were aged 18–88 years. There were no statistical differences in gender (*P* = 0.551) and age (*P* = 0.257) between the two groups (Table 1).

**Table 1:** Comparisons of gender and age between the two groups

Statistic indexes	The control group (n = 199)	The case group (n = 187)	Statistic value	*P* value
Gender [case (%)]			0.356	0.551
Male	89 (44.723)	78 (41.711)		
Female	110 (55.276)	109 (58.289)		
Age (years old, mean ± standard deviation)	53.34 ± 15.15	51.61 ± 14.82	1.134	0.257

### The occurrence and grade of air emboli

Among 199 cases in the control group, air emboli were discovered in 21 cases with an occurrence rate of 10.55%, including 11 cases with single air embolus and 10 cases with multiple air emboli. Among 187 cases in the case group, air emboli were observed in 7 cases (an occurrence rate of 3.74%), with single air embolus in 2 cases and multiple air emboli in 5 cases. Moreover, the cases of air emboli were less in the case group than in the control group with a statistical significance (*P* = 0.010, Table 2).

**Table 2:** The occurrence of air emboli in two groups of patients

Statistic indexes	The control group (n = 199)	The case group (n = 187)	Statistic value	*P* value
Existence of air emboli (case)			6.645	0.01
Yes	21	7		
No	178	180		

The 21 cases of air emboli in the control group consisted of 15 cases of small-grade air emboli, 6 cases of moderate-grade air emboli, and no cases of large-grade air emboli. The 7 cases of air emboli in the case group comprised 7 cases of small-grade air emboli and no cases of moderate- and large-grade air emboli. Cases with air emboli in the case group were less than those in the control group, and only small-grade air emboli were monitored in the case group (Table 3).

**Table 3:** Grading results of grading of air emboli (the Fisher’s exact test) in the two groups

Statistic indexes	The control group (n = 21)	The case group (n = 7)
Grading (case)		
Small grade	15	7
Middle grade	6	0

### The number, location, and diameter of air emboli

**Fig. 4:**
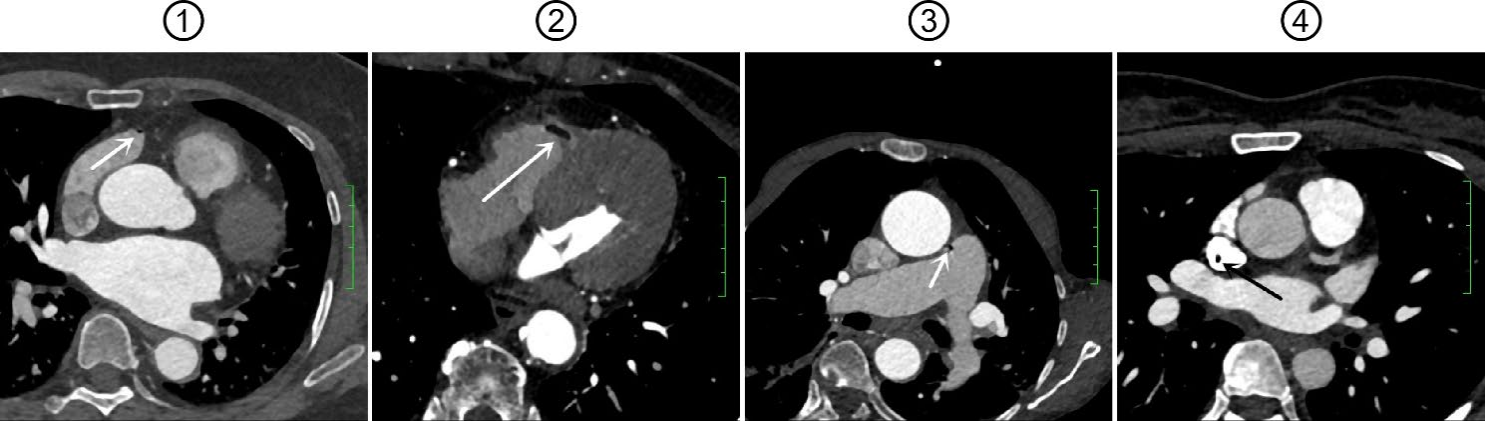
Representative patients Notes: ① A bubble-shaped air embolus in the right atrium (arrow) as shown by CCTA examination; ② A bubble-shaped air embolus in the right ventricle (arrow) as shown by CCTA examination; ③ A bubble-shaped air embolus in the main pulmonary artery (arrow) as shown by CCTA examination; ④ A bubble-shaped air embolus in the superior vena cava (arrow) as shown by CCTA examination. CCTA, coronary computed tomography angiography

A total of 42 air emboli were observed in 21 cases of air emboli from the control group, with a diameter of 0.8–11.8 mm [mean: (1.950 ± 1.719) mm], including 17 air emboli in the right atrium, 14 air emboli in the right ventricle, 8 air emboli in the main pulmonary artery, and 3 air emboli in the superior vena cava (Fig. 4). In 7 cases of air emboli from the case group, there existed 12 air emboli with a diameter ranging from 1.0 to 3.5 mm [mean: (1.608 ± 0.727) mm], among which 5 air emboli in the right atrium, 4 air emboli in the right ventricle, 3 air emboli in the main pulmonary artery, and no air emboli in the superior vena cava. The differences in diameter (*P* = 0.559) and location (*P* = 0.673) of air emboli were not statistically significant between the two groups (Table 4).

**Table 4:** The number, location (the Fisher’s exact test), and diameter (Mann-Whitney rank-sum test) of air emboli in two groups

Statistic indexes	The control group (n = 42)	The case group(n = 12)	Statistic value	*P* value
Mean diameter(mm)	1.950 ± 1.719	1.608 ± 0.727	-0.585	0.559
Location (case)			1.545	0.673
The right atrium	17	5		
The right ventricle	14	4		
The main pulmonary artery	8	3		
The superior vena cava	3	0		

## Discussion

Venous air emboli are most commonly found in all kinds of punctures, injections, and surgeries, in the process of diagnosis and treatment [13]. When a certain amount of air reaches the right heart along the blood flow, rapid cardiac impulses can mix the blood and air to form foamy blood bubbles, which may fill the cardiac chamber to hinder the venous blood flow, resulting in severe circulatory disturbance [14]. Moreover, the entry of the foamy blood bubbles to the pulmonary artery branch contributes to air embolisms in the lung [15]. For patients with patent foramen ovale, air may enter the artery via oval foramen from the vein and lead to air embolism in the artery [16]. If air exactly enters the coronary artery, even only 0.5 mL of air can result in myocardial ischemia, triggering severe consequences such as myocardial infarction [17–19]. These findings suggest that the severity of VAE is determined by the location and amount of air entering the blood. However, the lethal amount of air emboli has not reached an agreement. The extensively accepted lethal amount is over 100 mL in China [20] and 200–300 mL abroad [21].

CCTA images of patients (199 cases in the control group and 187 cases in the case group) after contrast agent injections were analyzed. The following results were obtained: (1) The control group had an occurrence rate of venous air emboli of 10.55%, in accordance with previous reports (7–23%) [3–5]. Additionally, an occurrence rate of venous air emboli of 3.74% was observed in the case group, lower than the occurrence rate of venous air emboli in previous literature. The occurrence rate of venous air emboli in the case group was decreased as compared to that in the control group (*P* = 0.010), which indicated that our method was capable of lowering the occurrence rate of venous air emboli in the process of tube connection during CTA examination. (2) There were only 7 cases of small-grade venous air emboli in the case group, whilst there were 15 cases of small-grade venous air emboli and 6 cases of moderate-grade venous air emboli in the control group. Grades of venous air emboli were fewer, and no air bubble images of moderate grade and large grade existed in the case group compared with the control group. (3) The diameter of venous air emboli was decreased from 1.950 ± 1.719 mm in the control group to 1.608 ± 0.727 mm in the case group. Despite no statistical significance (*P* = 0.559), the diameter of venous air emboli was reduced on the whole in the case group in comparison with the control group. (4) The number of venous air emboli was 42 and 12 in the control and case groups, respectively. Venous air emboli were frequently observed in the right atrium of both groups, concordant with most literature [11, 22]. The number of venous air emboli in each location did not differ between the two groups (*P* = 0.673).

The formation of air emboli may be attributed to the following factors: (1) The operations of the puncture of 18 G indwelling needles are nonstandard, resulting in the entry of small air bubbles into the human body. (2) Even though the frequently used high-pressure injectors are equipped with an air sensing system, they are still not sensitive to the infinitesimal air bubbles and cannot timely warn, which contributes to the possible situation that small air bubbles enter the human body. (3) Air may be formed due to gaps during connection of each tube (connection between the internal tube and the external tube of the high-pressure injector, connection between the external tube and the extension tube of the high-pressure injector, and so on) [8, 11, 13, 22].

The aforesaid issues can be resolved from the following aspects. (1) The training and management of nursing staff should be strengthened, and operations must be conducted in strict accordance with the procedures to avoid human errors as far as possible. (2) The insensibility of the high-pressure injectors to the infinitesimal air bubbles cannot be effectively solved at present. Nevertheless, it is believed that the improvements and upgrades of machines will finally solve this problem in the future. (3) Our research mainly investigated methods of reducing air production during tube connection, and it was verified by our results that the extra addition of a set of flush tubes to drain 20–30 mL liquids may be an efficient method of diminishing the occurrence rate of air emboli introduced in tube connection during CTA examination. Differences in the grade and size of venous air emboli were not statistically significant between the two groups, which may be attributable to the essentially relatively low occurrence rate of air emboli. Consequently, different results will be obtained if the sample size is enlarged in the future. Different from our method, Jia et al. used the method of adding the step of preflushing the power injector [6], which also was effective in reducing the incidence of VAE. However, the study of Jia et al. unraveled that there existed moderate-grade air emboli in the control and preflushing groups, whereas our study exhibited no moderate-grade air emboli in the control and case groups. This difference may be related to different methods and sample sizes.

There are several limitations in this research. Due to the wholly low occurrence rate of venous air emboli, the relatively small sample size in our research may result in a deviation in the statistical conclusions, which merits further research with a larger sample size in the future. This research only included patients receiving CCTA examination, and the clinical follow-up observations were not arranged for the patients with venous air emboli.

In a word, this modified method of saline test injection (a set of operations of tube flushing and liquid drainage are added in the conventional saline test injection) is one of the effective solutions to reduce the occurrence rate of venous air emboli. This modified method is feasible and practically significant. The following research will further explore the causes and resolutions of the occurrence of venous air emboli. More precautions should be taken for the venous air emboli to avoid the potential risks, and techniques of preventing air from entering vessels to the greatest extent are still necessary.

## Data Availability

The datasets used or analyzed during the current study are available from the corresponding author on reasonable request.
